# A spatial analysis for geothermal energy exploration using bivariate predictive modelling

**DOI:** 10.1038/s41598-021-99244-6

**Published:** 2021-10-05

**Authors:** Andongma W. Tende, Mohammed D. Aminu, Jiriko N. Gajere

**Affiliations:** 1 Department of Geology, Kano University of Science and Technology, Wudil, Kano, Nigeria; 2 Department of Petroleum Chemistry, American University of Nigeria, Yola, Nigeria; 3 Department of Geology, Nasarawa State Polytechnic, Lafia, Nigeria

**Keywords:** Environmental sciences, Solid Earth sciences, Energy science and technology

## Abstract

The development of predictive maps for geothermal resources is fundamental for its exploration across Nigeria. In this study, spatial exploration data consisting of geology, geophysics and remote sensing was initially analysed using the Shannon entropy method to ascertain a correlation to known geothermal manifestation. The application of statistical index, frequency ratio and weight of evidence modelling was then used for integrating every predictive data for the generation of geothermal favourability maps. The receiver operating/area under curve (ROC/AUC) analysis was then employed to ascertain the prediction accuracy for all models. Basically, all spatial data displayed a significant statistical correlation with geothermal occurrence. The integration of these data suggests a high probability for geothermal manifestation within the central part of the study location. Accuracy assessment for all models using the ROC/AUC analysis suggests a high prediction capability (above 75%) for all models. Highest prediction accuracy was obtained from the frequency ratio (83.3%) followed by the statistical index model (81.3%) then the weight of evidence model (79.6%). Evidence from spatial and predictive analysis suggests geological data integration is highly efficient for geothermal exploration across the middle Benue trough.

## Introduction

Nigeria’s energy supply is in a deplorable condition and requires urgent attention to meet the increasing needs of population rise. Since the 1950s, Nigeria has been heavily dependent on fossil fuels and hydro-electric power as the foremost source of energy and by 2015 a total of 4000 MW was generated for a population of over 170 million^1^. In recent years, energy development policies have often emphasized on diversification in energy resources with specific interest on renewable energy such as solar, wind, biomass and geothermal^2,3^. Among these energy sources, geothermal is one of the most promising as it appears to dominate major geological terrains around the world. Also, there have been a gradual but steady increase in geothermal energy production in many countries due to technological advancement in the areas of exploration, upstream engineering, power production and direct use^4^. Nevertheless, geothermal energy has been credited for its environmental friendliness, although zones of potential occurrence are often linked to natural and environmental disasters such as earthquakes^5,6^.

The exploration and exploitation of Nigeria’s geothermal resources is a viable task due to the widespread availability of enormous prospects that occur mostly within the Cretaceous sedimentary basins as well as certain parts of the basement complex terrains. Evidence from recent studies have suggested widespread geothermal prospects in the form of volcanic plugs, mud volcanoes and/or thermal springs^7^. More so, deduced geothermal gradient for most oil wells within the Niger Delta basin have revealed a significant geothermal potential^8,9^. The successful exploration for these resources remains a key criterion for effectively implementing a switch into geothermal energy utilisation. Although a wide range of exploration tools are available for geothermal prospecting, previous exploration strategies within Nigeria have been largely centred on field measurements and geophysical data interpretations^10–12^. Also, satellite mapping of hydrothermal alteration associated with geothermal prospects was explored by Abubakar et al.^13^. Usually, the identification of hydrothermal signatures related to geothermal prospects must be interpreted with caution since these altered minerals may also point to zones of significant mineral occurrence^14^. However, for the effective management of geothermal exploration programs, it is imperative to integrate spatial data as a means of identifying the most suitable location and extent for further study or survey^15,16^.

## Geological setting

**Figure 1:**
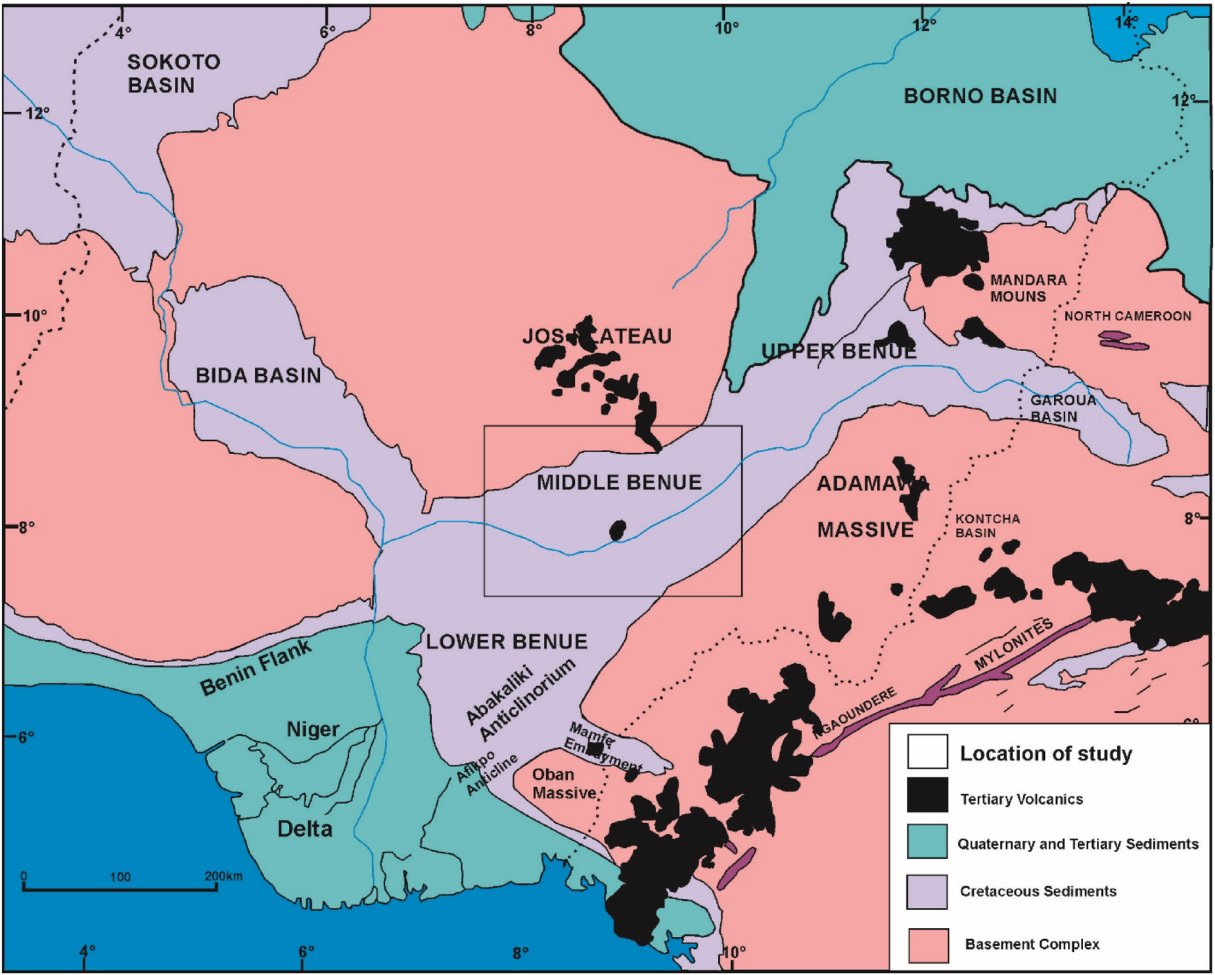
Regional geology and location of the study area (modified after Benkhelil^31^).

**Figure 2:**
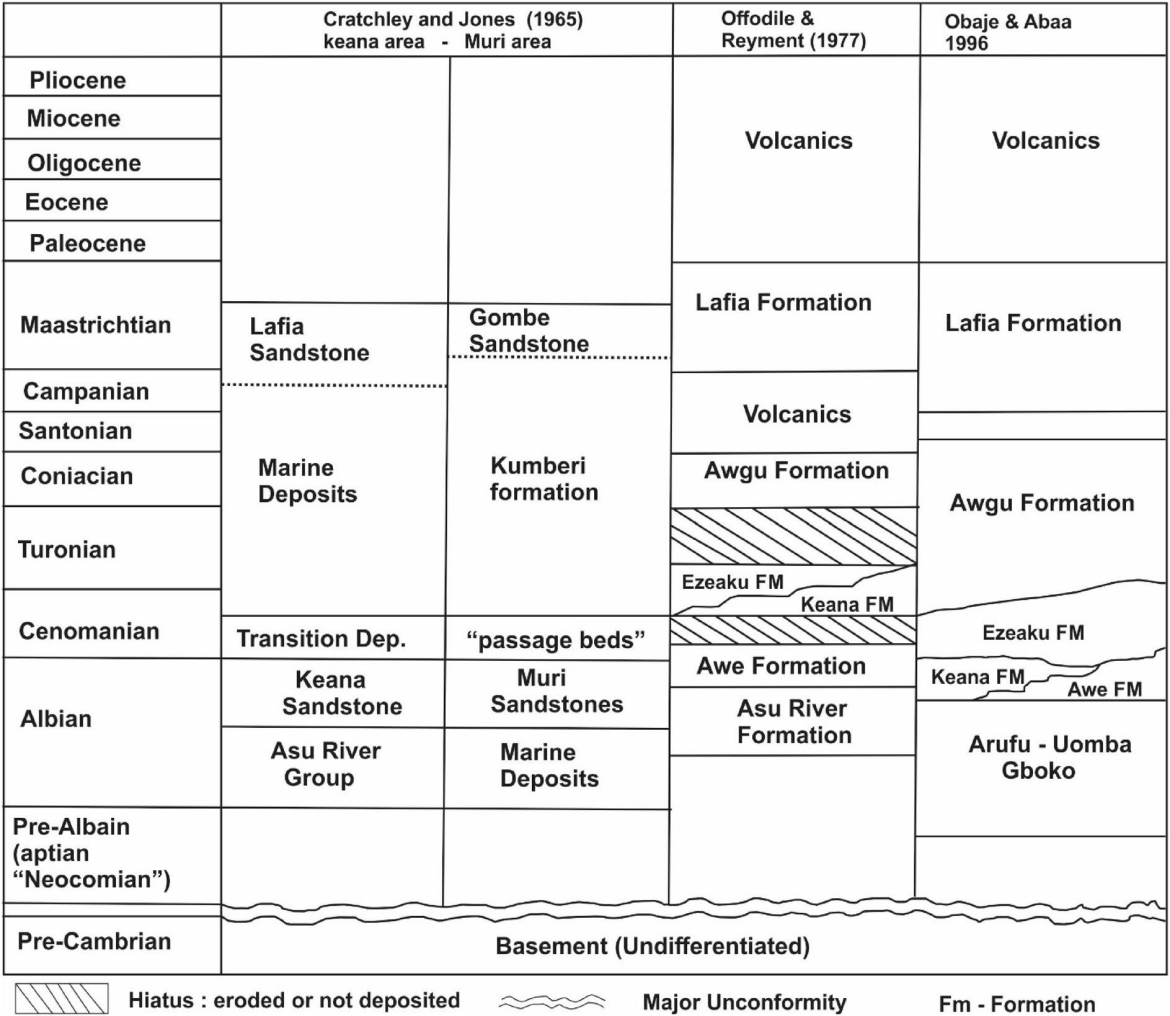
Regional stratigraphy of the mid Benue trough.

The study location (Fig. 1) which forms part of the mid Nigerian Benue trough is geographically situated within latitude N7.4° to N9° and longitude E7.7° to E10.1°. The geology as described by Obaje et al.^17^, can be subdivided into six stratigraphical units consisting of the Asu River group, the Eze Aku formation (Awe formation, Keana formation, Makurdi formation), the Awgu and the Lafia sedimentary units (Fig. 2). The Asu River group represents the oldest sediments, deposited from the mid to late Albian marine transgression of the south Atlantic and Gulf of Guinea. In terms of lithology, this group is considered a marine deposit that consist of limestones, shales and calcareous shales, micaceous siltstones, mudstones and fine grained sandstones^18^. Based on previous studies, the Asu River group is considered fossiliferous with numerous indications of ammonites, agglutinated and foraminiferal taxa reported by Offodile and Reyment^19^ and Peters^20^. Within the study location, outcrops of the Asu River group have been reported on the crest of the Keana Anticline and the eastern section of Keana town Offodile and Reyment^19^. Basically, the Asu River group is overlain by transitional beds of the Awe formation which is regarded as a passage or transitional beds and characterised by a dominant flaggy whitish and medium to coarse grained calcareous sandstones, siltstones, carbonaceous shales and clays^18,19^. According to Ariyo^21^ the occurrence of sandstone units within this formation is characterised by a continuous finning associated with increasing micaceous content towards the basal part of the formation. Generally, the Awe formation represents a late Albian to early Cenomanian regression that have been traced over large extent of the Benue trough. In the Awe town, an estimated of 100 m for the Awe formation have been reported by Offodile and Reyment^22^. Stratigraphically, this formation is overlain by the Keana formation, which is considered to be products of Cenomanian regression^23^ and consist essentially of cross bedded fine to very coarse grained and conglomeritic gritty/arkose sandstones of fluvial and deltaic origin^19,24^. Around Keana-Awe area estimated thickness of 800 m of the this formation has been reported by Ofoegbu^25^. Outcrops of the Keana formation are more common on the eastern and western flanks of the Keana anticline. The Eze Aku formation overlays the Keana formation and consist mainly of grey to black shales with clay horizons in association with fine to medium grained sandstones and limestones^26^. According to Salako et al.^27^, the Eze Aku formation was deposited during a late marine transgression that occurred in the Cenomanian period. Around Makurdi area, the Eze Aku formation interfingers with the Awgu formation^23^. Generally, the Awgu formation is which overlays the Eze Aku formation is considered to represent an abrupt termination of marine sedimentation within the mid Benue trough^28^. This process is evidenced by persistent occurrence of carbonaceous and calcareous shales, shaley limestones, sandstones, siltstones and coal beds^28^. The Awgu formation which has been dated as Turonian to Coniacian (or Early Santonian)^29^. Offodile and Reyment^19^ reported the existence of numerous intercalations between the continental and shallow marine conditions. This is defined by an interbedded occurrence of coal seams with shale, limestone and occasional occurrence of sandstones^30^. The Lafia formation is generally considered the youngest sedimentary deposits within the Mid Nigerian Benue trough^29^, and is defined by the presence of poorly consolidated ferruginous sandstones (which are often cross bedded) in association with flaggy mudstones and clay containing paleosol horizon^19^. Ferruginous features associated with the Lafia formation are indicative of a syn-depositional origin affected by strong oxidising conditions^19^. According to Obaje et al.^29^, the Lafia formation are mainly continental (fluviatile) deposits of Maastrichtian age. Generally the thickness of the Lafia formation hardly exceeds 50 m, although estimated thickness of 500 – 1500 m have been proposed by Offodile and Reyment^22^.

## Materials

### Target dataset

Geological occurrence of hot springs and hydrothermal fluids in wells and boreholes are often considered as geothermal manifestations^5,6,15,32^. In the Awe area and mid Nigerian Benue trough, these occurrence have been previously reported^7,30^. Information on geothermal data was gathered from the open literature^30^, and from field studies. A geological field survey lasting one week was undertaken within the study location. During field survey, the GPS (Global Positioning System) longitude and latitude co-ordinates of geothermal springs were recorded on a field notebook. The main guide for locating un-discovered geothermal manifestations were dependent on intrinsic knowledge of local field guides. Upon completion of fieldwork, the co-ordinates for these occurrence obtained from field survey and the open literature were entered into a Microsoft Excel spreadsheet where they were converted into point data using the ArcMap 10.3. The point data were used for predicting and validating geothermal energy occurrence across the study location.

### Exploration dataset

**Figure 3:**
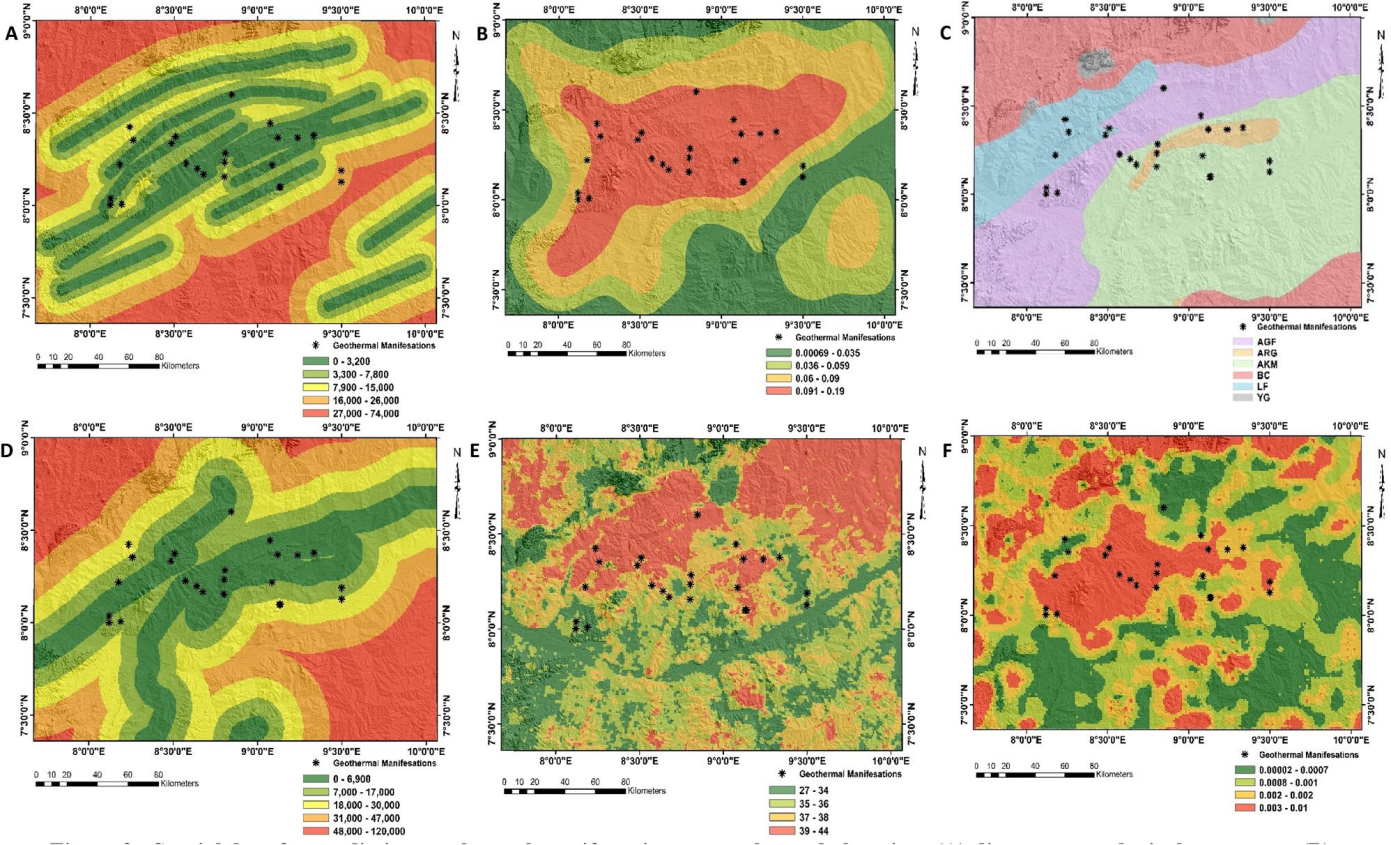
Spatial data for predicting geothermal manifestation across the study location: (**A**) distance to geological structures, (**B**) lineament density, (**C**) lithological units, (**D**) distances to sedimentary contacts, (**E**) surface temperature, and (**F**) gravity data.

Four exploration data which include structural, lithological, geophysical and land surface temperature data were integrated for identification of geothermal prospective zones within the mid Benue trough (Fig. 3). According to Brehme et al.^33^, Cashman et al.^34^, Faulds et al.^35^ and Husein et al.^36^, geological structures display a considerable influence on geothermal systems by providing possible pathways for migration of hydrothermal fluids across the subsurface environment^37^. Structural data for evaluating geothermal potential within the study area consist of distances from favourable structures and structural density. The generation of these data include a manual digitization of lineaments from high resolution digital elevation data, followed by spatial proximity analysis using the Euclidean distance tool. Also, the Kennel density algorithm was effectively applied for generating density patterns of structures across the study location. Lithological control on geothermal systems is based on the porosity and permeability of the host rock^38,39^. Within the Awe area, host rock lithologies is defined by several sedimentary formations intruded by numerous volcanic dykes^40^. Information on lithological data was obtained from an existing geological map published by the Nigerian Geological Survey Agency^41^. The lithological data was subset and screen digitized using ArcMap 10.3. Additional lithological information used for evaluating geothermal resources consist of distances to sedimentary contacts. The manual digitization of sedimentary contacts was carried out followed by a Euclidean analysis to generate a rasterized distance to contact image. Generally, gravity methods are considered cost effective and reliable for investigating geothermal systems in many regions across the world^42^. In this study, bouguer corrected gravity data obtained from National Geospatial Intelligence Agency^43^ was processed for identification of geothermal potential zones. The ‘xyz’ data was imported into Oasis montaj 8.4 software interface where it was subjected to the analytical signal filtering^44^. Surface temperatures obtained from satellite data are invaluable sources for mapping geothermal resources. Geothermal energy generated and stored within the earth’s crust is most likely to be transferred to the surface by conduction or convection of groundwater that forms hydrothermal systems^45,46^. On the surface, thermal anomalies resulting from subsurface geothermal activities can be mapped with thermal bands of satellite systems. The MODIS (Moderate Resolution Imaging Spectroradiometer) thermal band (MOD 11) taken from https://earthexplorer.usgs.gov was used for mapping geothermal activities within the middle Benue trough. The digital numbers were converted to degree Celsius using Eq. (1):

1 $$LST = \left( {DN*0.02} \right) - 273.1$$

where $LST$ represents the land surface temperature and $DN$ is the digital numbers in pixels.

## Methods

**Figure 4:**
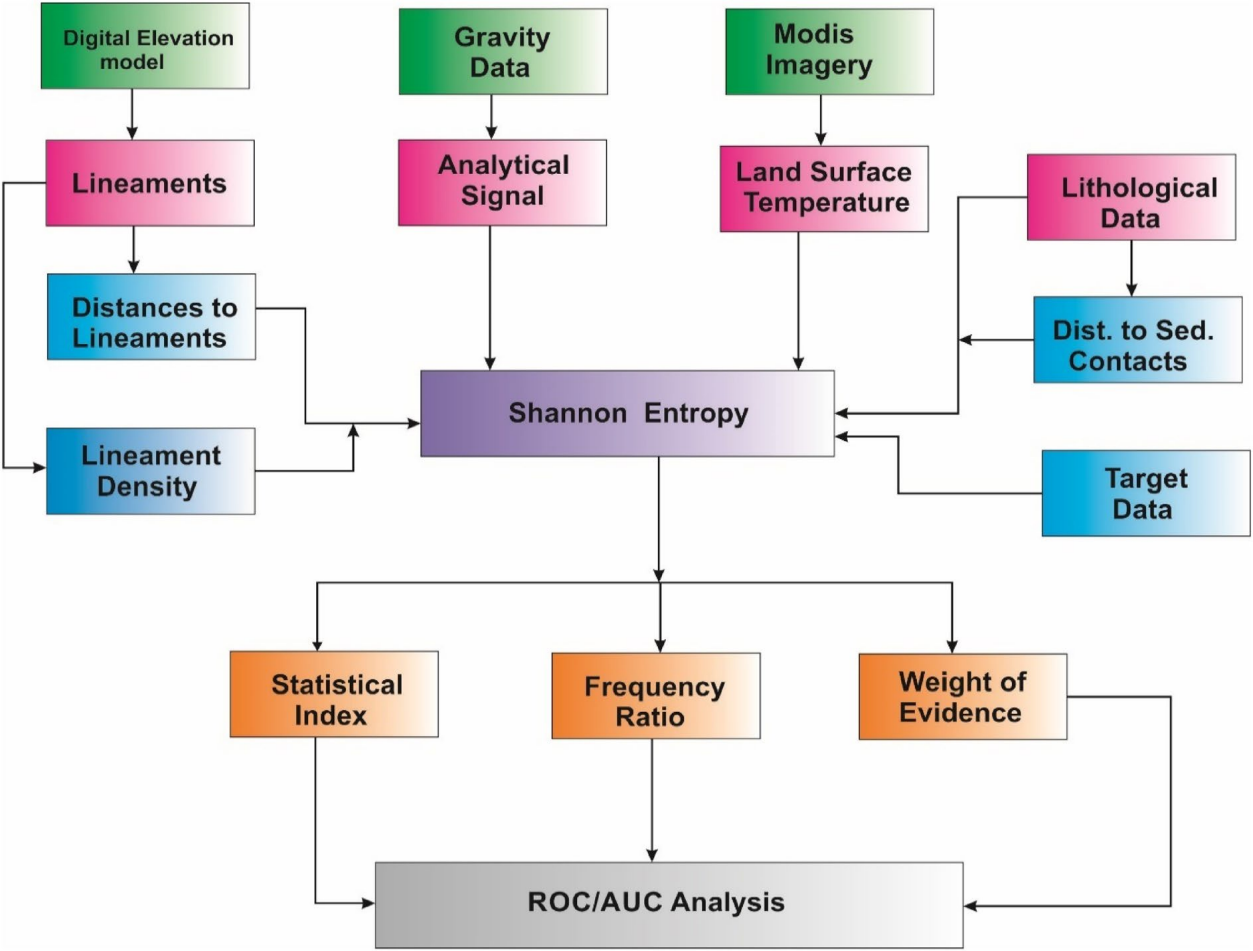
Flowchart illustrating the methods used in the study.

The prediction for geothermal energy was implemented systematically using a series of methods and procedures as shown in Fig. 4. Exploration dataset depicting geological structures (lineaments) were first extracted from digital elevation model and further processed by generating data pertaining to Euclidean distances to favourable geological structures and structural intensity defined as lineament density. Similarly, analytical signal algorithm applied to Bouguer gravity data was used for enhancing geophysical signatures related to gravity, while information on land surface temperature was generated from MODIS satellite data. Lithological data and distances to sedimentary contacts were extracted from existing geological maps. With the aid of target data (geothermal manifestations), the Shannon entropy was used to determine the predictive significance for all exploration data. After Shannon entropy analysis, exploration data with very low predictive significance were eliminated while those with a considerable level of significance were further integrated by application of statistical index, frequency and weight of evidence model. The receiver operating/area under curve (ROC/AUC) analysis was used to evaluate the reliability of every predictive model.

### Shannon entropy analysis

The statistical uncertainty associated with spatial data structure can be effectively evaluated using the Shannon entropy technique^47^. In most cases, these uncertainties can be expressed as the average expected value of information contained in a message and can be defined as the negative log of probability distribution on outcome^48^. Basically, the application of Shannon entropy in predictive modelling and feature selection can be implemented using Eqs. (2) to (6):

2 $$E_{ij} = \frac{FR}{{\mathop \sum \nolimits_{j = 1}^{mj} FR}}$$

where FR = frequency ratio, $E_{ij}$ = probability density for each class.

3 $$H_{j} = - \mathop \sum \limits_{i = 1}^{{m_{i} }} E_{ij} logE_{ij} ,\quad j = 1 \ldots n$$

4 $$H_{jmax} = log_{2} M_{j} , M_{j - number\,of\,classes}$$

5 $$I_{j} = \left( {H_{jmax} - H_{jmax} } \right), I = \left( {0,1} \right)j = 1$$

6 $$V_{j} = I_{j} FR$$

where $H_{j}$ and $H_{jmax}$ represents the entropy values, while $I_{j}$ denotes the information coefficient. $M_{j}$ signifies the number of classes within each exploration data and $V_{j}$ is the derived weighting from 0 to 1.

### Statistical index (SI)

The statistical index (SI) model is widely applied in predictive mapping^49^. This method initially proposed by^50^ is defined as the natural log of event density within a categorical class divided by the event density for the whole area^51,52^. Statistically, the SI model can be illustrated using Eq. (7).

7 $$W_{i} = \ln \left( {\frac{{D_{ij} }}{D}} \right) = ln\left[ {\left( {\frac{{N_{ij} /S_{ij} }}{N/s}} \right)} \right]$$

where, $W_{i}$ is the weight computed for a given class $i$, while $D_{ij}$ represents the density of geothermal occurrence (number of geothermal manifestations per unit area) for a given class $i$ within a specific parameter $j$ and $D$ is the total geothermal density for the entire area.

A higher positive value for computed SI indices suggest a possible relationship between the independent variables and a given categorical unit within a given condition factor^53^. Negatively computed SI values may suggest the insignificant ability of a defined class in predicting a given event^54^.

### Frequency ratio (FR)

Generally, the application of FR in statistical modelling is based on the presumption that the future occurrence of events are more likely under a similar condition to those of the past^55^. In most cases, the FR model estimates and evaluates the probabilistic relationship existing between the dependent and an independent variable^56,57^. In this study, FR for each parameter was computed by dividing the ratio of event occurrence on the area ratio. This computation can be expressed statistically using Eq. (8).

8 $$FR = {\text{ln}}\left( {\frac{Class\,Density }{{map\,Density }}} \right)$$

where class density indicates the number of geothermal evidence per unit area within a given class and map density suggest the number of geothermal energies per unit area for the total area of interest. The class density and map density can be computed using the Eqs. (9) and (10):

9 $$class\, density = \frac{{N_{pix} \left( {S_{i} } \right)}}{{N_{pix} \left( {N_{i} } \right)}}$$

10 $$map\, density = \frac{{\mathop \sum \nolimits_{i = 1}^{n} N_{pix} \left( {S_{i} } \right)}}{{\mathop \sum \nolimits_{i = 1}^{n} N_{pix} \left( {N_{i} } \right)}}$$

where $N_{pix} \left( {S_{i} } \right)$ is the total number of pixels with geothermal evidence within class $i$, and $N_{pix} \left( {N_{i} } \right)$ is the total number of pixels in class $i$.

$\sum\nolimits_{i = 1}^{n} {N_{pix} } \left( {S_{i} } \right)$ is the total number of pixels representing geothermal occurrence for the entire area and $\sum\nolimits_{i = 1}^{n} {N_{pix} } \left( {N_{i} } \right)$ total pixels for the entire area.

According to Tehrany et al.^58^, Cao et al.^59^, computed FR value above 1 suggest a stronger association between the dependent and independent variables, while values below 1 may imply a weaker relationship.

### Weight of evidence (WoE)

The spatial proximity between specific classes of a given variable and point data representing discrete events can be adequately evaluated using the WoE analysis^60^. According to Lee et al.^61^, the WoE employs the log linear form of Bayesian probability in ascertaining the statistical relationship between a dependent and one or several independent variables. The basic implementation of WoE analysis can be attained by calculating the positive weight (W^+^) and negative weight (W^−^) of class attributes within a given variable^62^. Positive weight attributes describe a derived weight within the test domain while the negative weighting attributes signifies weighting out of the test domain^63^. The positive and negative weighting for each class is computed using Eqs. (11) and (12):

11 $$W_{i}^{ + } = ln\frac{{P\left\{ {B/A} \right\}}}{{P\left\{ {B/\overline{A}} \right\}}}$$

12 $$W_{i}^{ + } = ln\frac{{P\left\{ {\overline{B}/A} \right\}}}{{P\left\{ {\overline{B}/\overline{A}} \right\}}}$$

where $P$ is the probability and $ln$ is the natural log. $B$ and $\overline{B}$ are indications of presence and non-presence of the independent variable, while $A$ and $\overline{A}$ suggest the existence and non-existence of an event.

Positive weight $(W_{i}^{ + } )$ evaluates the influence exhibited by a given class on a defined event, while the negative weighting $(W_{i}^{\_} )$ quantifies the influence on a given event due to the absence of a factor.

Usually, a contrast value is often computed to optimize the spatial association between the independent and dependent variable. Equation (13) is usually employed to determine the contrast between the positive and negative weights.

13 $$C_{w} = W_{i}^{ + } + W_{i}^{\_}$$

However, the elimination of discrepancies resulting from scenarios associated with smaller number of events and area of a given class could be achieved by computing the studentised contrast using Eq. (14).

14 $$C_{s} = {\raise0.7ex\hbox{$C$} \!\mathord{\left/ {\vphantom {C {s\left( C \right)}}}\right.\kern-\nulldelimiterspace} \!\lower0.7ex\hbox{${s\left( C \right)}$}}$$

where $C_{s}$ is the studentised contrast, $C$ is the contrast and $s\left( C \right)$ is the standard deviation of the contrast. Values of $s\left( C \right)$ are usually computed using Eq. (15).

15 $$s\left( C \right) = \sqrt {S^{2} \left( {W^{ + } } \right) + S^{2} \left( {W^{ - } } \right)}$$

### Receiver operating curve/Area under curve

Statistical validation of every predictive model are essential attributes for demonstrating its reliability and augmenting the confidence for real life applications. In this study, the ROC and AUC validation methods were effectively used in ascertaining the reproducibility of all predictive models^64^. According to Mas et al.^65^, the ROC method permits performance assessment via a binary classification system alongside a ranking order or a continuous output values. Most often, the ROC curve is generated by utilizing binary curve that compares the sensitivity (True negative rate) against 1-specificity (False positive rate)^66^. The false positive rate (FPR) denotes the proportion of negative pixels incorrectly classified by the classifier, while the true positive rate (TPR) is a measure of negative pixel proportion correctly classified. Generally, FPR and TPR can be computed using Eqs. (16) and (17).

16 $$FPR = \frac{FP}{{\left( {FP + TN} \right)}}$$

17 $$TPR = \frac{TP}{{\left( {TP + FN} \right)}}$$

where: $FP$ represents the false positive or incorrect prediction of positive classes, $TN$ denotes the true negative or correct prediction of negative classes, $TP$ illustrates the true positive or correct prediction of positive classes, $FN$ represents the false negative or incorrect prediction of negative classes.

The quantitative estimation of prediction accuracy for every model can be achieved by calculating its AUC value. Generally, AUC outputs are represented by probabilistic values ranging from 0 to 1. An AUC value of 1 suggests complete success of the classifier while an AUC value of 0.5 shows randomness in the classification scheme.

## Results

### Statistical correlation

The statistical correlation among exploratory variables is presented on Table 1. This matrix suggests a generally low positive correlation among existing variables. However, a significant positive correlation of 0.56 was observed for lineament density and temperature dataset. Similarly, most variables also exhibited a generally low negative correlation trend with significant negative correlation observed for lineament density, distances to the ENE-WSW lineaments temperature dataset. These variables had a correlation value of -0.65 and -0.408, respectively.

**Table 1:** Statistical correlation among exploratory datasets.

Variables	Geology	Lin. Den	Temp	Geophysics	ENE-WSW Dist	Sed. Contacts
Geology	1	0.009	− 0.083	0.194	− 0.034	− 0.119
Lin. Den	0.009	1	0.557	− 0.139	− 0.657	− 0.335
Temp	− 0.083	0.557	1	− 0.251	− 0.408	0.103
Geophysics	0.194	− 0.139	− 0.251	1	− 0.170	− 0.300
ENE-WSW Dist	− 0.034	− 0.657	− 0.408	− 0.170	1	0.374
Sed. Contacts	− 0.119	− 0.335	0.103	− 0.300	0.374	1

### Analysis of variable significance

**Figure 5:**
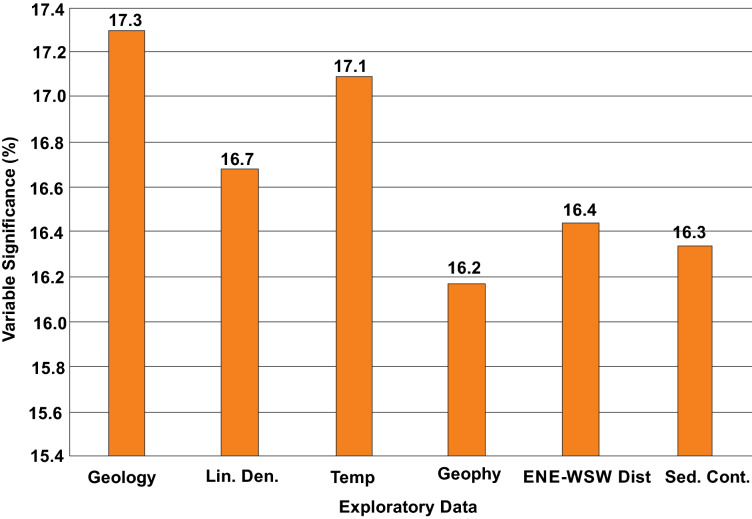
Variable influential analysis for exploration data.

Fig. 5 illustrates a variable significance analysis for all exploration data used in the study. Analysis of this data suggest geological, lineament density and temperature are the most influential parameters for prospectivity mapping of geothermal energy within the study locations. These spatial datasets displayed percentile influence of 17.3, 16.7 and 17.1, respectively. However, the least influential factors were geophysics, distances to ENE-WSW lineaments and distances to sedimentary contacts. These datasets had a percentile influence of 16.2, 16.4 and 16.3, respectively.

### Application of statistical index (SI)

Table 2 illustrates weights generated from all exploration parameters using the SI model. Based on these weights, a positive spatial correlation is observed for geothermal occurrence and lithological units consisting of the Lafia formation, Awe-Keana formation and Asu River group. However, a negative spatial association is observed between geothermal occurrence and the Awgu formation. No spatial association was observed for crystalline rocks and geothermal occurrence. Also, SI modelling applied to lineament density revealed a positive spatial association with lineament density above 0.0905. Below this range, all other classes displayed negative spatial association with known occurrence of geothermal resources. SI modelling for temperature variations across the study location reveals a positive spatial association with temperatures above 35.93 ºC. Below this value, a general negative spatial association is observed with known geothermal occurrence. Analysis of spatial association of geothermal occurrence between geophysical signatures suggest a positive relationship at magnetic intensity value above 0.1 × 10^2^ nT. However, magnetic values below this intensity generally exhibit a negative spatial association within known geothermal occurrence. An evaluation of distance to ENE-WSW lineaments suggests a positive association with geothermal occurrence at proximal distances less than 3.2 km. Above this distance, there is no spatial association with geothermal occurrence. The distance to sedimentary contacts displayed a positive spatial association with geothermal occurrence at distances below 17 km, with a negative spatial association at distances from 18 – 30 km. Above 30 km, no spatial association in geothermal occurrence is observed for distances to sedimentary contacts.

**Table 2:** Parameter weighting by application of statistical index.

Data	Classes	SI
Geology	Basement Complex	0
	Younger Granite	0
	Lafia Fm	0.748
	Awgu Fm	− 0.068
	Awe-Keana-Makurdi Fm	0.014
	Asu River Group	1.815
Lin. Den	0.000689–0.0347	− 1.824
	0.0348–0.0593	− 1.847
	0.0594–0.0904	− 0.747
	0.0905–0.185	1.172
Temp	27.05–34.2	− 1.154
	34.2–35.93	− 0.053
	35.93–38.04	0.113
	38.04–44.4	0.481
Geophysics	2.4 × 10^−5^–6.7 × 10^−4^	− 1.794
	6.7 × 10^−4^–1.0 × 10^−3^	− 0.793
	1.0 × 10^−3^–1.5 × 10^−3^	0.429
	1.5 × 10^−3^–9.6 × 10^−3^	0.571
Dist. to Lin	0–3,200	1.257
	3,300–7,800	− 0.025
	7,900–15,000	− 1.627
	16,000–26,000	− 0.929
	27,000–74,000	0
Contacts	0–6,900	1.045
	7,000–17,000	0.168
	18,000–30,000	− 0.025
	31,000–47,000	0
	48,000–120,000	0

### Application of frequency ratio (FR)

Statistical computation of weights using the FR is shown in Table 3. Its application for predicting geothermal occurrence is defined by a positive spatial association with lithological units such as the Lafia formation, Awgu formation, Awe-Keana-Makurdi formation and the Asu River group. However, no spatial association was observed for the crystalline basement rocks. Also, spatial prediction with lineament density suggests an overall positive association for all parameter classes with predictive class above 0.0905 exhibiting the highest spatial association with geothermal occurrence. The correlation of geothermal occurrence with temperature suggests a positive association for all parameter class with temperature corresponding to values above 38.4 ºC, being the most correlating. For geophysical data, magnetic signatures displayed a positive association with every parameter class. However, magnetic signatures above 0.1 × 10^2^ nT appears to be more associated with geothermal occurrence. Spatial association with distances to ENE-WSW lineaments suggest a positive association for all parameters with distances less than 3.3 km exhibiting the most significant association. For distances to contact, a positive association was observed for all classes with distances below 30 km. Above this distance, no correlation was observed for parameter data.

**Table 3:** Parameter weighting by application of frequency ratio.

Data	Classes	FR
Geology	Basement Complex	0
	Younger Granite	0
	Lafia Fm	2.112
	Awgu Fm	0.934
	Awe-Keana-Makurdi Fm	1.014
	Asu River Group	6.142
Lin. Den	0.000689–0.0347	0.161
	0.0348–0.0593	0.158
	0.0594–0.0904	0.474
	0.0905–0.185	3.229
Temp	27.05–34.2	0.315
	34.2–35.93	0.948
	35.93–38.04	1.119
	38.04–44.4	1.618
Geophysics	2.4 × 10^−5^–6.7 × 10^−4^	0.166
	6.7 × 10^−4^–1.0 × 10^−3^	0.453
	1.0 × 10^−3^–1.5 × 10^−3^	1.536
	1.5 × 10^−3^–9.6 × 10^−3^	1.771
Dist. to Lin	0–3,200	3.514
	3,300–7,800	0.975
	7,900–15,000	0.197
	16,000–26,000	0.395
	27,000–74,000	0
Contacts	0–6,900	2.842
	7,000–17,000	1.183
	18,000–30,000	0.975
	31,000–47,000	0
	48,000–120,000	0

### Application of weight of evidence (WoE)

The analysis of spatial association of exploration data with geothermal occurrence using the weight of evidence (WoE) model was interpreted using the studentized contrast statistics. Analysis of the data from Table 4, suggest a positive spatial relationship with geothermal occurrence were observed for the Lafia formation, Awe-Keana formation and Asu River group lithologies. However, a negative correlation was observed for the Awgu formation and the crystalline basement rocks. Spatial relationship between geothermal occurrence and lineament density parameter was defined by a positive spatial association at lineament intensity value above 0.095. Below this value, all parameters displayed a negative spatial relationship with geothermal occurrence. For the temperature parameter, a positive relationship was observed for classes with temperature value above 35.93 ºC, with all other classes displaying a negative relationship. The geophysical data revealed a positive relationship with geothermal occurrence at magnetic intensity above 0.1 × 10^2^ nT. Parameter classes with magnetic intensity below this value exhibited a general negative relationship with known geothermal occurrence. At distances less than 3.2 km from the ENE-WSW lineaments, a positive association with geothermal occurrence is observed. All distances above 3.2 km exhibited a general negative relationship with geothermal occurrence. Also, the distances to sedimentary contacts were defined by a positive association at distances below 17 km, with a significant spatial association observed for distances below 6.9 km. Parameter classes with distances from 17 – 47 km displayed a negative spatial relationship, while a null relationship was observed for classes above 47 km.

**Table 4:** Parameter weighting by application of weight of evidence.

Data	Classes	W^+^	W^−^	C	Std. C
Geology	Basement Complex	0	0.01	− 0.01	− 0.016
	Younger Granite	0	0.099	− 0.099	− 0.157
	Lafia Fm	0.748	0.074	0.674	1.066
	Awgu Fm	− 0.068	0.227	− 0.295	− 0.467
	Awe-Keana-Makurdi Fm	0.014	− 0.484	0.498	0.788
	Asu River Group	1.815	− 0.174	1.99	3.147
Lin. Den	0.000689–0.0347	− 1.822	0.251	− 2.072	− 1.602
	0.0348–0.0593	− 1.845	0.25	− 2.095	− 1.62
	0.0594–0.0904	− 0.745	0.156	− 0.9	− 0.696
	0.0905–0.185	1.175	− 1.609	2.784	2.153
Temp	27.05–34.2	− 1.15	0.207	− 1.357	− 2.539
	34.2–35.93	− 0.049	0.012	− 0.061	− 0.114
	35.93–38.04	0.117	− 0.046	0.163	0.305
	38.04–44.4	0.485	− 0.511	0.996	1.863
Geophysics	2.4 × 10^−5^–6.7 × 10^−4^	− 1.779	0.262	− 2.041	− 2.486
	6.7 × 10^−4^–1.0 × 10^−3^	− 0.778	0.169	− 0.947	− 1.154
	1.0 × 10^−3^–1.5 × 10^−3^	0.443	− 0.23	0.673	0.82
	1.5 × 10^−3^–9.6 × 10^−3^	0.586	− 0.58	1.166	1.419
Dist. to Lin	0–3,200	1.259	− 0.91	2.169	2.279
	3,300–7,800	− 0.023	0.004	− 0.027	− 0.028
	7,900–15,000	− 1.625	0.185	− 1.81	− 1.902
	16,000–26,000	− 0.927	0.136	− 1.063	− 1.117
	27,000–74,000	0	0	0	0
Contacts	0–6,900	1.047	− 0.595	1.641	3.265
	7,000–17,000	0.17	− 0.045	0.215	0.428
	18,000–30,000	− 0.023	0	− 0.023	− 0.046
	31,000–47,000	0	0.219	− 0.219	− 0.435
	48,000–120,000	0	0	0	0

### Predictive modelling

**Figure 6:**
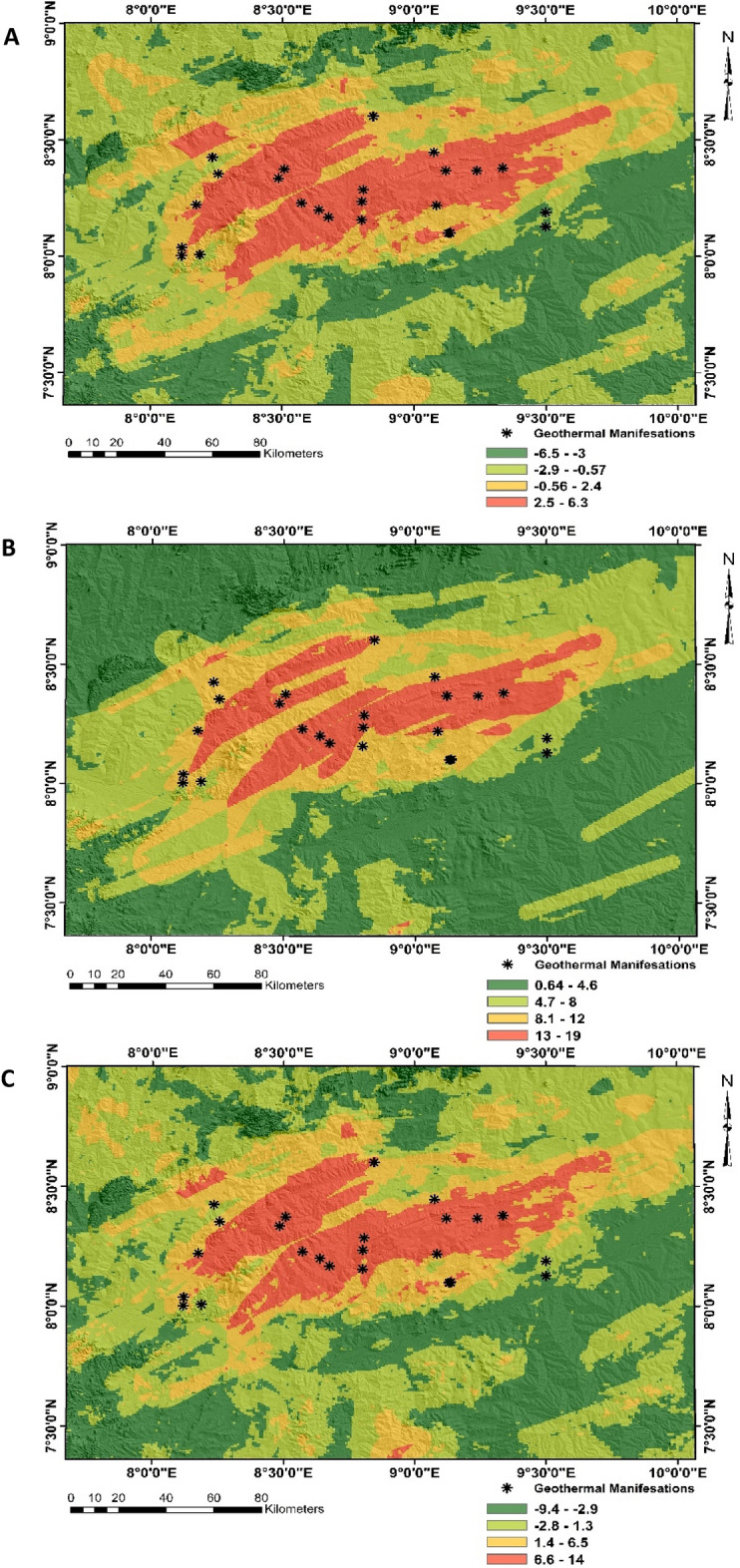
Geothermal predictive maps: (**A**) application of statistical index, (**B**) application of frequency ratio, (**C**) application of weights of evidence.

**Figure 7:**
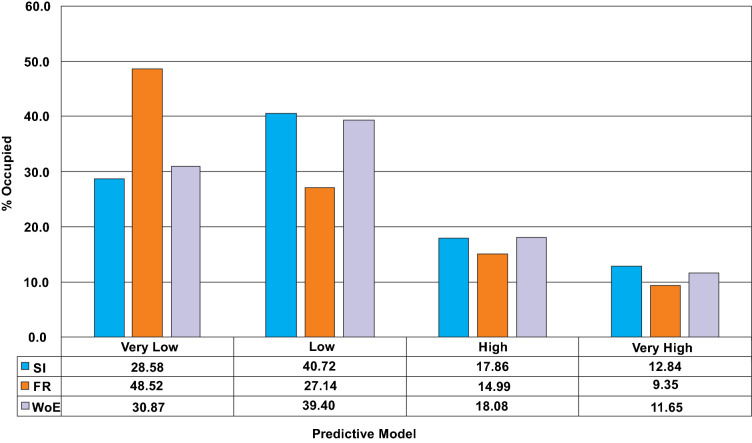
Percentile distribution of predictive class within each model.

Figure 6 illustrates the geothermal predictive maps based on SI, FR and WoE models. Visual analysis of these maps suggests a generally high potential for geothermal resources around the central axis of the study area while zones of low potentials are more common around the peripheries. However, the predicted zones appear to be more extensive within the SI and model when compared to those of FR model. An analysis of percentile statistics for every class within each predictive model is presented on Fig. 7. A visual summary of these statistics suggests the very low potential class is defined by a percentile extent of 28.58%, 48.52% and 30.87% for the SI, FR and WoE model. However, percentile summary of the low predictive class suggests a percentile extent of 40.72%, 27.14% and 39.40% for the SI, FR and WoE model. The high predictive class are generally characterized by percentage summary of 17.86% for the SI, 14.99% for FR and 18.08% for WoE model. For the very high predictive class, a percentage extent of 12.84% was recorded for the SI while 9.53% and 11.65% were recorded for the FR and WoE model.

### Model validation

**Figure 8:**
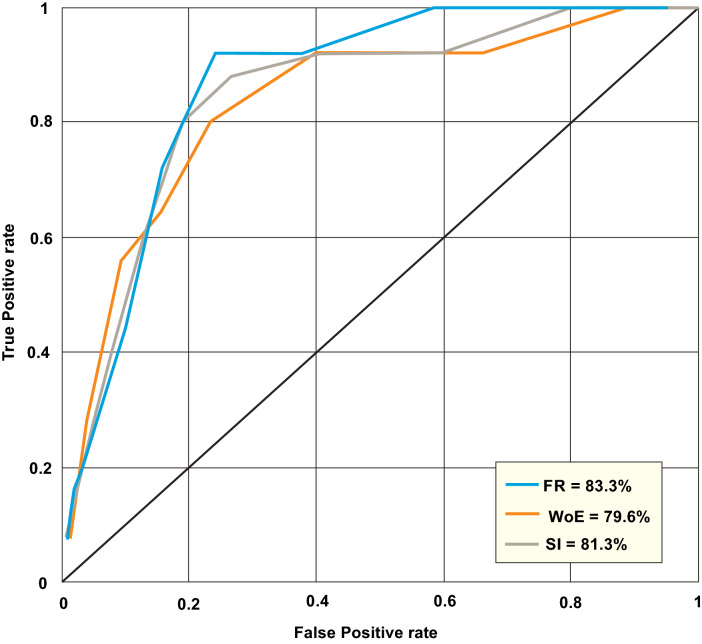
Statistical validation for geothermal predictive models.

Accuracy assessment for all predictive models is illustrated in Fig. 8. Based on this plot, a high prediction accuracy above 75% was envisaged for all models. Statistical validation with the ROC/AUC method suggests the FR model had the best accuracy for predicting geothermal resources. Basically, accuracy level with the FR model were approximately 83.3%. The next best accuracy was obtained from the SI model with an accuracy level of 81.3. The WoE had the least prediction accuracy at 79.6%.

## Discussions

Previous studies on geothermal exploration in Nigeria^67,68^ have often accentuated on the need for a more robust approach in geothermal exploration. The application of digital mapping technology by geospatial data integration offers a more sustainable approach for exploring geothermal resources due to its immense accuracy, cost effectiveness and high reliability^16,39^. Within Nigeria, this technology remains relatively new and highly fascinating since it can deduce spatial patterns that may point to geothermal manifestations. The successful implementation of this method is often backed by an in-depth knowledge on existing relationship between exploration data and known geothermal occurrence^69^. The variable influential plot shown on Fig. 5 represents a more assertive tool for unravelling the spatial complexities between geothermal occurrence and exploration data and reveals the effectiveness of every parameter in geothermal mapping. A concise analysis of this plot suggests an augmented relationship exists between known geothermal points and spatial data related to lithological and structural intensity. Within the Nigerian Benue trough, evidence for lithological and structural control of geothermal occurrence have been reported by Musa et al.^7^, and a substantial correlation of geothermal resources with lineament intensity may point to the possible presence of numerous closely spaced intersecting faults^70^. A positive correlation with land surface temperature obtained from thermally calibrated MODIS imagery may suggest a near surface manifestation for geothermal occurrence across the study location. This is evidenced by the numerous and widespread occurrence of hot springs and volcanic rocks^30,40^. Generally, a visual analysis of geothermal predictive maps generated by all three predictive models (SI, FR and WoE) suggest a high potential within the central axis of the study location.

This zone coincides with the occurrence of Keana anticline. According Babalola^71^ the high potential of geothermal occurrence around several fold belts within the Nigerian Benue trough can be attributed to the possible emplacement of magmatic intrusions. Statistical evaluation of accuracy level using the ROC/AUC plots suggest a significant prediction level above 70%. However, geothermal predictions with the FR models appears to be more reliable when compared to SI and WoE models. The discrepancies in accuracy levels for all predictive models can be attributed to a general complexity of the spatial models that amplifies and affects the interaction between the input data and their associated error on the output models^72^. The ENE-WSW trend assumed by high geothermal potential zones also correlates with directional trends for intrusion structures^73^.

## Conclusions

The study entails a regional exploration survey for geothermal resources using spatial data integration. Bivariate statistical analysis was employed to ascertain the most probable zones for geothermal resource occurrence. Based on spatial correlation, the existence of geothermal resources within the Nigerian Benue trough is effectively controlled by geological attributes related to structures and lithologies. These features displayed a high spatial correlation within known geothermal occurrence. Based on spatial analysis, the central axis represented the most probable zones for district scale exploration of geothermal resources. The high prediction accuracy for all models (> 75%) suggest an excellent reliability that may be invaluable for future geothermal exploration.
